# Comparison of Crestal Bone Loss and Peri-Implant Clinical Outcomes Between Platform-Switched and Platform-Matched Dental Implants: A Prospective Observational Clinical Study

**DOI:** 10.7759/cureus.113640

**Published:** 2026-07-30

**Authors:** Pritha Negi, Deepak Yadav, Sree Ram Subba R Gudimetla, Annesha K Konwar, Sanpreet S Sachdev, Saritha Maloth

**Affiliations:** 1 Department of Prosthodontics, Crown, and Bridge, K. M. Shah Dental College and Hospital, Sumandeep Vidyapeeth (Deemed to be University), Vadodara, IND; 2 Department of Oral and Maxillofacial Surgery, Shree Guru Gobind Singh Tricentenary University, Gurugram, IND; 3 Department of Oral and Maxillofacial Surgery, Priyadarshini Dental College and Hospital, Thiruvallur, IND; 4 Department of Prosthodontics, Government Dental College, Silchar, IND; 5 Department of Oral Pathology and Microbiology, Bharati Vidyapeeth (Deemed to be University) Dental College and Hospital, Mumbai, IND; 6 Department of Dentistry, Koppal Institute of Medical Sciences, Koppal, IND

**Keywords:** alveolar bone loss, dental implant-abutment design, dental implants, longitudinal studies, peri-implant tissue

## Abstract

Introduction

Preservation of the crestal bone is essential for the long-term success of dental implants. Platform switching has been proposed to minimize marginal bone loss by shifting the implant-abutment interface away from the crestal bone and improving stress distribution. This study aimed to compare the crestal bone loss and peri-implant clinical outcomes between platform-switched and platform-matched dental implants over a 12-month follow-up period.

Materials and methods

This prospective longitudinal observational study included 90 patients undergoing implant-supported rehabilitation, with 45 patients receiving platform-switched and 45 receiving platform-matched implants. Clinical and radiographic evaluations were performed at baseline and 6 and 12 months after prosthetic loading. Crestal bone loss was measured using standardized digital intraoral periapical radiographs. The secondary outcomes included probing depth, modified bleeding index, and modified plaque index. Data were analyzed using appropriate statistical tests and analysis of covariance, with statistical significance set at p < 0.05.

Results

Baseline demographic and clinical characteristics were comparable between the groups (p > 0.05). At 12 months, the platform-switched group demonstrated significantly lower crestal bone loss than the platform-matched group (0.80 ± 0.30 mm vs. 1.18 ± 0.37 mm; p < 0.001). Bone loss at six months was also significantly lower in the platform-switched group (0.49 ± 0.18 mm vs. 0.72 ± 0.22 mm; p < 0.001). The platform-switched group showed significantly lower probing depth (2.57 ± 0.36 mm vs. 3.02 ± 0.62 mm; p < 0.001) and modified bleeding index (0.53 ± 0.33 vs. 0.90 ± 0.37; p < 0.001), whereas modified plaque index did not differ significantly between groups (p = 0.080). The implant platform design remained an independent predictor of crestal bone preservation after covariate adjustment.

Conclusion

Platform-switched implants demonstrated superior crestal bone preservation and improved peri-implant soft tissue health compared to platform-matched implants after 12 months of functional loading. These findings support the use of platform-switched implant designs to enhance peri-implant tissue stability and reduce marginal bone remodeling.

## Introduction

Dental implants have become the preferred treatment modality for replacing missing teeth owing to their high survival rates, predictable functional outcomes, and ability to preserve oral health and patient quality of life. Despite the excellent long-term success of implant therapy, preservation of the peri-implant crestal bone remains one of the most important determinants of implant longevity, esthetic stability, and long-term functional success [1,2]. Early marginal bone loss may compromise soft tissue support, increase the risk of peri-implant disease, and ultimately affect the prognosis of implant-supported restorations [3]. Consequently, considerable research has focused on identifying implant design features and clinical protocols that minimize crestal bone remodeling following implant placement and functional loading.

Marginal bone remodeling around dental implants is a multifactorial process influenced by surgical trauma, implant-abutment microgap, occlusal loading, biologic width establishment, implant positioning, bone quality, and patient-related systemic factors [4,5]. Among these variables, the implant-abutment interface has received particular attention because inflammatory cell infiltration around the microgap has been suggested as a potential contributor to crestal bone resorption [6]. This has led to the development of the platform-switching concept, in which the diameter of the prosthetic abutment is intentionally smaller than that of the implant platform [7]. By moving the implant-abutment junction inward, platform switching is believed to reduce stress concentration at the crestal bone, shift the inflammatory infiltrate away from the bone crest, and promote better preservation of peri-implant hard and soft tissues [8].

Several clinical studies and systematic reviews have reported that platform-switched implants may demonstrate reduced crestal bone loss compared to conventional platform-matched implants [7,9]. However, the magnitude of this benefit remains inconsistent across studies owing to differences in implant systems, patient characteristics, surgical techniques, prosthetic protocols, follow-up durations, and outcome assessment methods. Moreover, most available evidence has originated from randomized clinical trials or retrospective analyses, whereas prospective observational studies reflecting routine clinical practice remain comparatively limited [10]. Therefore, further evidence generated under real-world clinical conditions is necessary to better understand the effectiveness of platform switching and its influence on peri-implant tissue health.

This study aimed to compare the clinical performance of platform-switched and platform-matched dental implants with respect to crestal bone preservation and peri-implant tissue health following functional loading. Specifically, the study evaluated changes in crestal bone levels at 6 and 12 months; assessed peri-implant clinical parameters including probing depth, modified bleeding index, and modified plaque index at 12 months; and determined whether implant platform design independently influenced crestal bone loss after adjusting for relevant demographic and clinical confounding factors.

## Materials and methods

This prospective longitudinal observational study was conducted in the Department of Oral and Maxillofacial Surgery, Priyadarshini Dental College and Hospital, India, between January 2023 and December 2024. Patient recruitment was carried out from January 2023 to December 2023, and all participants were prospectively followed for 12 months after prosthetic loading, with scheduled clinical and radiographic evaluations at baseline, six months, and 12 months. The follow-up intervals were selected because the greatest peri-implant crestal bone remodeling typically occurs during the first year after prosthetic loading, particularly within the initial six months, owing to biologic width establishment and adaptation to functional loading [3]. As this was an observational study, no randomization or treatment allocation was performed. Patients underwent implant therapy as part of routine clinical care and received either platform-switched or platform-matched implants according to the clinical judgment of the treating implantologist, anatomical considerations, and prosthetic requirements. The study protocol was approved by the Institutional Ethics Committee of Priyadarshini Dental College and Hospital (PDCH/IEC/12/2022/46 dated December 18, 2022). Written informed consent was obtained from all participants prior to enrollment.

Study participants

Patients presenting to the Department of Oral and Maxillofacial Surgery for implant-supported rehabilitation of single or multiple missing teeth in the posterior maxilla or mandible were screened for eligibility. Participants aged 18 years or older with healed extraction sites of at least four months' duration, adequate alveolar bone volume to accommodate a standard implant (diameter ≥3.5 mm and length ≥8 mm) without simultaneous bone augmentation, sufficient interocclusal restorative space, satisfactory oral hygiene, and willingness to attend all scheduled follow-up visits were included. Patients with uncontrolled systemic diseases, previous radiotherapy involving the head and neck region, current or previous bisphosphonate therapy, active periodontal disease, untreated dental caries, heavy smoking (>10 cigarettes/day), severe parafunctional habits, such as bruxism, pregnancy, lactation, or inability to comply with the follow-up protocol were excluded from the study. Each participant contributed only one implant to the study. In patients requiring multiple implant-supported restorations, a single implant was selected for analysis to ensure independence of observations and to avoid clustering effects.

Sample size

The sample size was estimated using G*Power software (version 3.1.9.7; Heinrich Heine University Düsseldorf, Düsseldorf, Germany), based on previously published differences in crestal bone loss between platform-switched and platform-matched implant systems. Assuming a Cohen's d effect size of 0.67, two-sided alpha error of 0.05, and statistical power of 80%, a minimum sample of 36 participants per group was required [11]. To compensate for an anticipated attrition rate of approximately 20% and ensure adequate statistical precision, 90 participants were recruited, comprising 45 patients treated with platform-switched implants and 45 patients treated with platform-matched implants.

Surgical protocol

All implant surgeries were performed by a single experienced implantologist with more than five years of clinical experience following a standardized surgical protocol under local anesthesia using 2% lidocaine with 1:100,000 epinephrine. Following aseptic preparation of the surgical field, a mid-crestal incision with or without limited-release incisions was made according to the clinical situation, and a full-thickness mucoperiosteal flap was carefully reflected to expose the alveolar crest. Osteotomy preparation was performed using sequential drills according to the manufacturer's recommended drilling protocol under copious sterile saline irrigation to prevent thermal injury to the surrounding bone.

**Figure 1:**
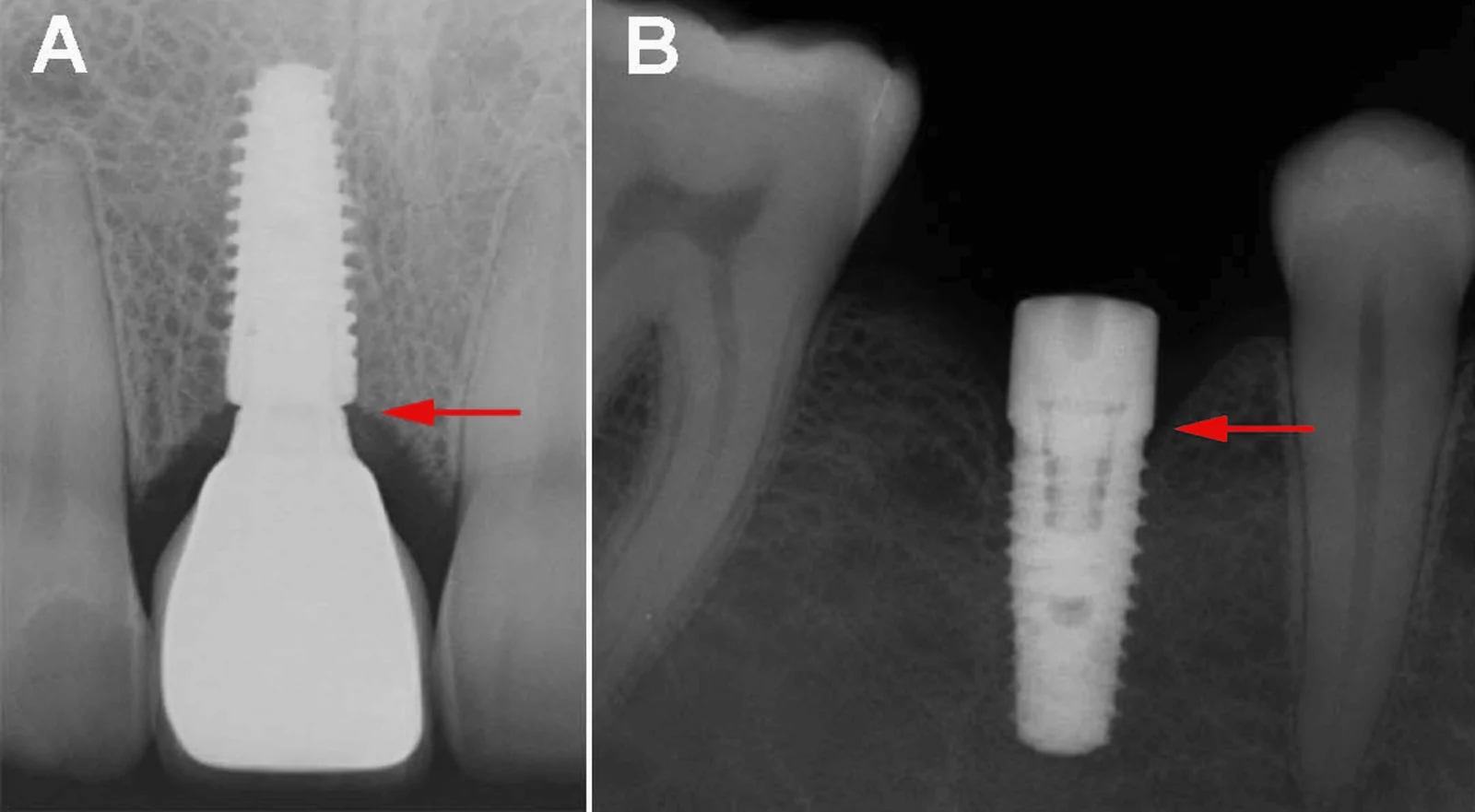
Intraoral periapical radiographs showing abutment type (red arrows) (A) Platform switch-type abutment in the maxillary central incisor region; (B) Platform match-type abutment in the mandibular first molar region. Images acquired from original patient data.

Patients received either Straumann Bone Level implants restored with platform-switched abutments (Institut Straumann AG, Basel, Switzerland) or Osstem TSIII implants restored with platform-matched abutments (Osstem Implant Co., Ltd., Seoul, South Korea). Implant grouping was based on the implant-abutment configuration rather than the implant system itself (Figure 1).

Implants were inserted at the crestal bone level using a calibrated surgical handpiece, and primary stability was clinically confirmed by insertion torque and implant stability assessment. All implants achieved adequate primary stability at placement and received healing abutments using a standardized transmucosal healing protocol. No implants required submerged healing with cover screws, thereby ensuring consistency in postoperative healing across all participants. The surgical site was irrigated thoroughly with sterile saline, and primary wound closure was achieved using interrupted 3-0 silk sutures. All participants received standardized postoperative instructions, including antibiotic therapy, analgesics, and 0.12% chlorhexidine mouth rinse, according to the departmental protocols. Sutures were removed after 10-14 days. Prosthetic rehabilitation was initiated after an osseointegration period of approximately three to four months in the mandible and four to six months in the maxilla, after which definitive implant-supported restorations were delivered.

Radiographic assessment

The primary outcome was crestal bone loss, assessed using standardized digital intraoral periapical radiographs obtained at definitive prosthetic loading (baseline) and 6 and 12 months after prosthetic loading. Radiographic measurements were independently performed by two calibrated implantologists (PN and DY) who were blinded to each other's measurements during the evaluation process. Images were analyzed using ImageJ software (version 1.54d; National Institutes of Health, Bethesda, MD, USA) after correcting for radiographic magnification. Marginal bone levels were measured from the implant platform to the first visible bone-to-implant contact on both the mesial and distal aspects of each implant, and the mean of the two measurements was considered for statistical analysis. Before the commencement of the study, both examiners underwent calibration using a representative set of radiographs that were not included in the final analysis. Intra-examiner and inter-examiner reliabilities were assessed using the intraclass correlation coefficient (ICC), and an ICC value greater than 0.90 was considered indicative of excellent agreement before formal data collection commenced.

Clinical examination

Clinical evaluations were performed by a single calibrated examiner (DY) at the time of definitive prosthetic loading (baseline) and at 6 and 12 months thereafter, using a standardized clinical protocol. Peri-implant tissue health was assessed by recording the probing depth, modified bleeding index, and modified plaque index using a calibrated UNC-15 periodontal probe (Hu-Friedy Mfg. Co., LLC, Chicago, IL, USA). All clinical measurements were obtained under standardized conditions to minimize examiner-related variability and ensure consistency throughout the follow-up period.

Statistical analysis

Statistical analysis was independently performed by a statistician (SSS) using SPSS Statistics for Windows, version 26.0 (IBM Inc., Armonk, NY, USA). Data distribution was assessed using the Shapiro-Wilk test. Continuous variables are expressed as mean ± standard deviation, whereas categorical variables are presented as frequencies and percentages. Baseline characteristics between the platform-switched and platform-matched cohorts were compared using the independent samples t-test for continuous variables and chi-square test for categorical variables. The effect of implant platform design on 12-month crestal bone loss was evaluated using analysis of covariance (ANCOVA), with implant type as the fixed factor, and age, implant length, implant diameter, jaw location, and bone quality were entered as covariates to control for potential confounding. Secondary clinical outcomes were analyzed using independent sample t-tests or Mann-Whitney U tests according to data distribution. A two-sided p-value of less than 0.05 was considered statistically significant for all analyses.

## Results

Ninety participants completed the study, with 45 patients (50.0%) in the platform-switched implant group and 45 patients (50.0%) in the platform-matched implant group.

Baseline characteristics

The baseline demographic and clinical characteristics of the two groups were comparable (Table 1). The mean age was 43.90 ± 10.10 years in the platform-switched group and 45.30 ± 10.20 years in the platform-matched group (p = 0.504). The platform-switched group comprised 24 (53.3%) males and 21 (46.7%) females, whereas the platform-matched group included 27 (60.0%) males and 18 (40.0%) females. The distribution of implant location, smoking status, diabetes mellitus, bone quality, implant length, and implant diameter did not differ significantly between groups (all p > 0.05), indicating good baseline comparability (Table 1).

**Table 1:** Baseline demographic and clinical characteristics of the study participants Data are presented as mean ± standard deviation (SD) for continuous variables and as number (percentage) for categorical variables. An independent samples t-test was used for continuous variables, and the chi-squared test was used for categorical variables. *p-value < 0.05 was considered statistically significant.

Characteristic		Group A platform-switched (n = 45)	Group B platform-matched (n = 45)	Test statistic	p-value	Effect size
Age, years	mean ± SD	43.90 ± 10.10	45.30 ± 10.20	t = 0.67	0.504	0.14
Sex, n (%)	Male	24 (53.3)	27 (60.0)	χ² = 0.18	0.671	0.05
	Female	21 (46.7)	18 (40.0)			
Jaw, n (%)	Maxilla	17 (37.8)	23 (51.1)	χ² = 1.12	0.289	0.11
	Mandible	28 (62.2)	22 (48.9)			
Smoking status, n (%)	Yes	11 (24.4)	10 (22.2)	χ² < 0.01	1.000	0.01*
	No	34 (75.6)	35 (77.8)			
Diabetes mellitus, n (%)	Yes	9 (20.0)	12 (26.7)	χ² = 0.25	0.618	0.05
	No	36 (80.0)	33 (73.3)			
Bone quality, n (%)	D1	3 (6.7)	3 (6.7)	χ² = 0.45	0.929	0.04*
	D2	18 (40.0)	17 (37.8)			
	D3	20 (44.4)	19 (42.2)			
	D4	4 (8.9)	6 (13.3)			
Implant length, mm	mean ± SD	10.10 ± 1.20	10.00 ± 1.20	t = 0.30	0.762	0.06
Implant diameter, mm	mean ± SD	4.10 ± 0.38	4.02 ± 0.43	t = 0.85	0.396	0.18

Crestal bone loss at 12 months

Twelve months after prosthetic loading, the platform-switched implant group demonstrated significantly lower crestal bone loss than the platform-matched group (Table 2). The mean crestal bone loss was 0.80 ± 0.30 mm in the platform-switched group compared with 1.18 ± 0.37 mm in the platform-matched group, representing a mean difference of 0.38 mm (95% CI: 0.24-0.52 mm). This difference was statistically significant (p < 0.001) with a large effect size (Cohen's d = 1.13).

**Table 2:** Comparison of crestal bone loss between platform-switched and platform-matched implants at 12 months Data are presented as mean ± standard deviation (SD). Group comparisons were performed using the independent samples t-test. Mean difference is presented with the 95% confidence interval (CI). A lower crestal bone loss (CBL) indicates better marginal bone preservation. *p-value < 0.05 was considered statistically significant.

Outcome	Group A platform-switched (n = 45)	Group B platform-matched (n = 45)	Mean difference (mm) (95% CI)	t value	p-value	Cohen d
CBL at 12 months, mm (mean ± SD)	0.80 ± 0.30	1.18 ± 0.37	0.38 (0.24 to 0.52)	5.33	< 0.001*	1.13

Bone loss over time

The evaluation of crestal bone remodeling over time demonstrated significantly lower marginal bone loss in the platform-switched group at both follow-up intervals (Table 3). mm in the platform-matched group (p < 0.001). At six months, the mean crestal bone loss was 0.49 ± 0.18 mm in the platform-switched group compared with 0.72 ± 0.22. At 12 months, bone loss increased in both groups but remained significantly lower in the platform-switched group (0.80 ± 0.30 mm) than in the platform-matched group (1.18 ± 0.37 mm) (p < 0.001), indicating superior preservation of marginal bone levels throughout the follow-up period.

**Table 3:** Comparison of crestal bone loss at 6 and 12 months after prosthetic loading Data are presented as mean ± standard deviation (SD). Between-group comparisons at each follow-up interval were performed using the independent samples t-test. *p-value < 0.05 was considered statistically significant.

Time point	Group A platform-switched (mean ± SD, mm)	Group B platform-matched (mean ± SD, mm)	t value	p-value	Cohen d
6 months post-loading	0.49 ± 0.18	0.72 ± 0.22	5.54	< 0.001*	1.14
12 months post-loading	0.80 ± 0.30	1.18 ± 0.37	5.33	< 0.001*	1.13

Peri-implant clinical parameters

A comparison of peri-implant clinical parameters at 12 months revealed significantly better peri-implant tissue health in the platform-switched group (Table 4). The mean probing depth was significantly lower in the platform-switched group (2.57 ± 0.36 mm) than in the platform-matched group (3.02 ± 0.62 mm) (p < 0.001). Similarly, the modified bleeding index was significantly lower in the platform-switched group (0.53 ± 0.33) than in the platform-matched group (0.90 ± 0.37) (p < 0.001). Although the modified plaque index was also lower in the platform-switched group (0.56 ± 0.27) than in the platform-matched group (0.68 ± 0.36), the difference was not statistically significant (p = 0.080).

**Table 4:** Comparison of peri-implant clinical parameters at 12 months Data are presented as mean ± standard deviation (SD). Between-group comparisons were performed using the independent samples t-test. *p-value < 0.05 was considered statistically significant.

Parameter	Group A platform-switched (n = 45)	Group B platform-matched (n = 45)	t value	p-value	Cohen d
Probing depth, mm (mean ± SD)	2.57 ± 0.36	3.02 ± 0.62	4.27	< 0.001*	0.89
Modified Bleeding Index (mean ± SD)	0.53 ± 0.33	0.90 ± 0.37	5.02	< 0.001*	1.05
Modified Plaque Index (mean ± SD)	0.56 ± 0.27	0.68 ± 0.36	1.77	0.080	0.38

Effect of implant platform design on crestal bone loss

After adjusting for potential confounding variables using ANCOVA, implant platform design remained a significant independent predictor of crestal bone loss at 12 months (Table 5). Platform-switched implants demonstrated a significantly lower adjusted crestal bone loss than platform-matched implants (p < 0.001). In contrast, age (p = 0.959), implant length (p = 0.411), and implant diameter (p = 0.087) did not significantly influence marginal bone loss. These findings indicate that the observed reduction in crestal bone loss was primarily attributable to the implant platform design rather than to the evaluated patient- or implant-related variables.

**Table 5:** Analysis of covariance evaluating the effect of implant platform design on crestal bone loss at 12 months Analysis of covariance (ANCOVA) was performed with implant type as the fixed factor and age, implant length, implant diameter, jaw location, and bone quality as covariates. *p-value < 0.05 was considered statistically significant. df - degrees of freedom

Source	Sum of squares	df	F value	p-value
Implant type (group)	2.937	1	26.36	< 0.001*
Age (covariate)	0.0003	1	0.003	0.959
Implant length (covariate)	0.076	1	0.68	0.411
Implant diameter (covariate)	0.333	1	2.99	0.087
Residual (error)	9.469	85	—	—

## Discussion

This prospective longitudinal observational study compared crestal bone preservation and peri-implant clinical outcomes between platform-switched and platform-matched implant systems over a 12-month follow-up period. The findings demonstrated that platform-switched implants exhibited significantly lower crestal bone loss and more favorable peri-implant soft tissue parameters than platform-matched implants. Even after adjustment for potential confounding variables, the implant platform design remained an independent predictor of marginal bone preservation, highlighting the potential biological advantage of the platform-switching concept.

Preservation of the marginal crestal bone is considered one of the most important determinants of long-term implant success because it contributes to the maintenance of peri-implant soft tissues, esthetic stability, and functional longevity. In the present study, the mean crestal bone loss at 12 months was significantly lower in the platform-switched group than in the platform-matched group. These findings are consistent with those reported by Rocha et al. [11], who demonstrated significantly reduced crestal bone loss around platform-switched implants in a three-year multicenter clinical trial. Similar observations were reported by Atieh et al. [12] in their systematic review and meta-analysis, which concluded that platform switching is associated with superior marginal bone preservation compared to conventional implant-abutment configurations. Therefore, the present findings provide additional prospective clinical evidence to support the biological benefits of platform switching under routine clinical conditions.

The findings of the present study are consistent with those of Guerra et al. [13], who, in a multicenter randomized clinical trial, reported significantly lower marginal bone loss around platform-switched implants than around platform-matched implants after one year of functional loading. The authors concluded that the platform-switching concept provides superior preservation of crestal bone levels, while maintaining high implant success rates. Several biological and biomechanical mechanisms may explain the improved bone preservation observed in the platform-switched implants. The implant-abutment microgap is displaced horizontally away from the crestal bone using an abutment with a smaller diameter than the implant platform. This inward repositioning may reduce bacterial infiltration and the associated inflammatory cell infiltration near the crestal bone while simultaneously relocating the stress concentration toward the central axis of the implant [14]. Finite element analyses have consistently demonstrated a lower crestal stress distribution in platform-switched implants, which may contribute to reduced marginal bone remodeling during functional loading [15]. Furthermore, preservation of the peri-implant biological width and improved soft tissue adaptation may limit crestal bone resorption.

The longitudinal assessment performed in this study showed that, although marginal bone remodeling occurred in both groups between 6 and 12 months, the platform-switched implants consistently demonstrated lower bone loss throughout the observation period. Early crestal remodeling during the first year following implant loading has been widely recognized as a physiological response related to the establishment of peri-implant soft tissue seal and adaptation to functional occlusal loading [16]. However, the significantly smaller magnitude of remodeling observed in the platform-switched group suggests that the implant-abutment design may substantially influence this biological adaptation process. Similar longitudinal trends have been described by Canullo et al. [17], who reported stable marginal bone levels around platform-switched implants during long-term follow-ups.

The peri-implant clinical parameters observed in the present study further support these radiographic findings. Patients treated with platform-switched implants demonstrated significantly lower probing depths and modified bleeding index scores than those receiving platform-matched implants, indicating healthier peri-implant soft tissues [18]. Reduced probing depth and bleeding on probing are generally regarded as indicators of stable peri-implant tissue attachment and reduced inflammatory activity. These findings are in agreement with previous investigations that reported improved peri-implant mucosal stability around platform-switched implants owing to better preservation of the biological width and more favorable connective tissue adaptation [10,12,19]. In contrast, although the modified plaque index was slightly lower in the platform-switched group, the difference was not significant. This finding suggests that plaque accumulation was primarily influenced by patient oral hygiene practices rather than implant platform configuration and emphasizes the importance of regular maintenance irrespective of implant design.

After adjustment for age, implant dimensions, jaw location, and bone quality, the implant platform design remained the only significant predictor of crestal bone preservation. Neither patient age nor implant dimensions significantly affected marginal bone loss. These findings indicate that the observed differences were predominantly attributable to the implant-abutment configuration rather than the baseline demographic or surgical variables.

Nevertheless, a previous systematic review reported no significant differences between platform-switched and platform-matched implants, particularly when different implant surfaces, loading protocols, or follow-up durations were evaluated [20]. Such discrepancies may be explained by variations in the study design, implant systems, prosthetic protocols, bone quality, sample size, and measurement methodologies. These observations are in agreement with systematic reviews by Annibali et al. [21] and Strietzel et al. [22], who highlighted the heterogeneity among clinical studies and emphasized that the benefits of platform switching may be influenced by multiple biological and prosthetic factors.

The findings of this study have several important clinical implications. Platform-switched implants may represent a preferable treatment option in situations where preservation of crestal bone and peri-implant soft tissue stability is particularly important, such as esthetically demanding regions or patients requiring long-term maintenance of peri-implant tissues. Improved marginal bone preservation may contribute to enhanced prosthetic stability, reduced biological complications, and improved long-term outcomes. Nevertheless, implant selection should always be individualized after considering patient-related factors, bone availability, prosthetic requirements, and the clinician's experience.

The present study had certain limitations. As an observational, non-randomized investigation, the possibility of residual confounding and selection bias cannot be completely excluded, despite statistical adjustment. The follow-up period was limited to 12 months after prosthetic loading, and therefore does not permit the assessment of long-term implant survival or peri-implant tissue stability. In addition, the study was conducted at a single center with a relatively modest sample size, which may limit the generalizability of the findings. Future multicenter randomized clinical trials with larger cohorts and longer follow-up periods are warranted to confirm the long-term advantages of platform switching and evaluate patient-reported outcomes and implant survival.

## Conclusions

Within the limitations of this prospective longitudinal observational study, platform-switched implants demonstrated significantly lower crestal bone loss and superior peri-implant tissue health than platform-matched implants after 12 months of functional loading. The implant platform design remained an independent predictor of marginal bone preservation after adjusting for potential confounding variables. These findings support the clinical use of platform-switched implants to enhance peri-implant bone stability and soft tissue health, although long-term multicenter studies are required to further validate these outcomes.
